# DAILY EXPERIENCES OF SOCIAL CONNECTION: REAL-WORLD ASSESSMENT STRATEGIES TO UNDERSTAND MECHANISMS

**DOI:** 10.1093/geroni/igae098.1877

**Published:** 2024-12-31

**Authors:** Kimberly Van Orden, Nancy Donovan

**Affiliations:** Chair; Discussant

## Abstract

Social connection is multifaceted construct that encompasses structural, functional, and subjective (quality) dimensions of relationships, all of which are associated prospectively with health, functioning, well-being, and longevity. Despite clear evidence linking social connection to health in later life, there are no clear evidence-based interventions and available community programs are under-utilized. One barrier to effectively intervening is the inability to tailor programs to person-specific contributors to disconnection. This symposium will describe findings from studies of social connection in later life that use innovative methods to examine person-specific and proximal causes, contributors, and consequences of social connection, including passive sensing via smartphones, analysis of social behavior (including speech patterns), and ecological momentary assessment. These methods allow for frequent measurement of indices of social connection in real-time (reducing recall bias) and in real-world contexts. Dr. Lee will present on the assessment of loneliness and social isolation among older adults using unstructured speech data in comparison to research surveys. Ms. Zhou will present on associations between sleep, social encounters, and momentary loneliness in older adults’ everyday life. Ms. VanBogart will present on associations between momentary loneliness and indices of stress (diurnal cortisol). Dr. Bower will present on momentary experiences of belonging in a sample of older adults with subjective cognitive decline. Dr. Van Orden will describe procedures and design considerations for EMA and passive sensing indices of social connection as outcomes in clinical trials to promote social connection. Dr. Donovan will facilitate a discussion to integrate findings and identify opportunities for future research.

